# Occurrence of Emery-Dreifuss muscular dystrophy in a rural setting of Cameroon: a case report and review of the literature

**DOI:** 10.1186/s13104-016-2363-1

**Published:** 2017-01-09

**Authors:** Cyril Jabea Ekabe, Jules Kehbila, Carlson–Babila Sama, Benjamin Momo Kadia, Martin Hongieh Abanda, Gottlieb Lobe Monekosso

**Affiliations:** 1 Grace Community Health and Development Association (GRACHADA), P.O Box 15, Kumba, Cameroon; 2 District Hospital Wum, Wum, North West Region Cameroon; 3 Galactic Corps Research Group (GCRG), Buea, South West Region Cameroon; 4 Bambalang Medicalised Health Centre, Bambalang, Northwest Region Cameroon; 5 Presbyterian General Hospital Acha-Tugi, Acha-Tugi, Northwest Region Cameroon; 6 Non Communicable Diseases Unit, Clinical Research Education, Networking & Consultancy (CRENC), Douala, Cameroon; 7 Department of Medicine, Faculty of Health Sciences, University of Buea and Global Health Dialogue Foundation, Buea, Cameroon

**Keywords:** Emery-Dreifuss muscular dystrophy, Case report, Rural setting, Cameroon

## Abstract

**Background:**

Emery-Dreifuss muscular dystrophy is a rare genetic muscular disease, presenting mainly with contractures, weakness and cardiac conduction abnormalities. Its clinical and laboratory similarities to other muscular dystrophies, and rarity poses diagnostic challenges, requiring a high index of suspicion in resource limited settings.

**Case presentation:**

An 8 year old sub-Saharan male presented with rigidity and deformity of both elbows and ankles, and weakness of the upper limbs and lower limbs for duration of 4 months. This progressed to inability to stand and walk. There was no mental impairment. Physical examination was remarkable for contractures of the elbows and ankles, and wasting of muscles of the limbs and trunk, with a scapulohumeroperoneal pattern, and tachycardia. After laboratory investigations, a diagnosis of Emery-Dreifuss muscular dystrophy was suspected. Physiotherapy was started, wheel chair was prescribed, and referral to a specialist center was done for appropriate management.

**Conclusions:**

Emery-Dreifuss muscular dystrophy is a rare disabling muscular disease which poses a diagnostic challenge. High index of suspicion is paramount for its early diagnoses to prevent orthopedic and cardiac complications. Prompt diagnosis and management is essential to improve on the prognosis of this disease.

## Background

Muscular dystrophy (MD) refers to a collective group of inherited, non-inflammatory muscular disorders resulting from genetic mutations. These mutations lead to a dysfunction in or deficiency of essential proteins vital in muscle cell stability with consequent progressive muscle degeneration without evidence of morphologic aberrations [1]. The occurrence of MD is quite rare with reported prevalence ranging between 19.8 and 25.1 per 100,000 person-years [2]. Amongst others, the varying disorders within the MD spectrum differ from each other based on the group of muscles affected, age at onset, severity of symptoms, pattern of inheritance and other organs affected [3].

Emery-Dreifuss muscular dystrophy (EDMD) is a rare genetic muscular disease with a worldwide incidence of 1 in 100,000 [3], presenting in late childhood and adulthood [4]. EDMD has three patterns: X-linked recessive, autosomal recessive, and autosomal dominant trends [5, 6]. Clinically, EDMD present with early contractures (involving the elbows, Achilles tendon and posterior cervical muscles), progressive weakness and atrophy with a scapulohumeroperoneal distribution, and cardiac conduction defects, myopathy or both [7, 8].

Muscular biopsy analysis, muscle enzymes assay, electro-myography, immunopathology and genetic studies play vital roles in the diagnosis of this pathology [8]. The clinical and laboratory similarities of EDMD to other muscular dystrophies couple with its rarity pose a diagnostic challenge. However, early diagnosis of EDMD is pivotal in preventing early mortality and morbidity from cardiac complications and muscular contractures respectively. Genetic screening of family members is also important to help identify family members at risk [9]. We herein to the best of our knowledge report the first case of EDMD in rural Cameroon presenting at late childhood with orthopedic complications as a result of delayed diagnosis, and impending cardiac complications necessitating early cardiac interventions so as to avert sudden cardiac death.

## Case presentation

**Fig. 1:**
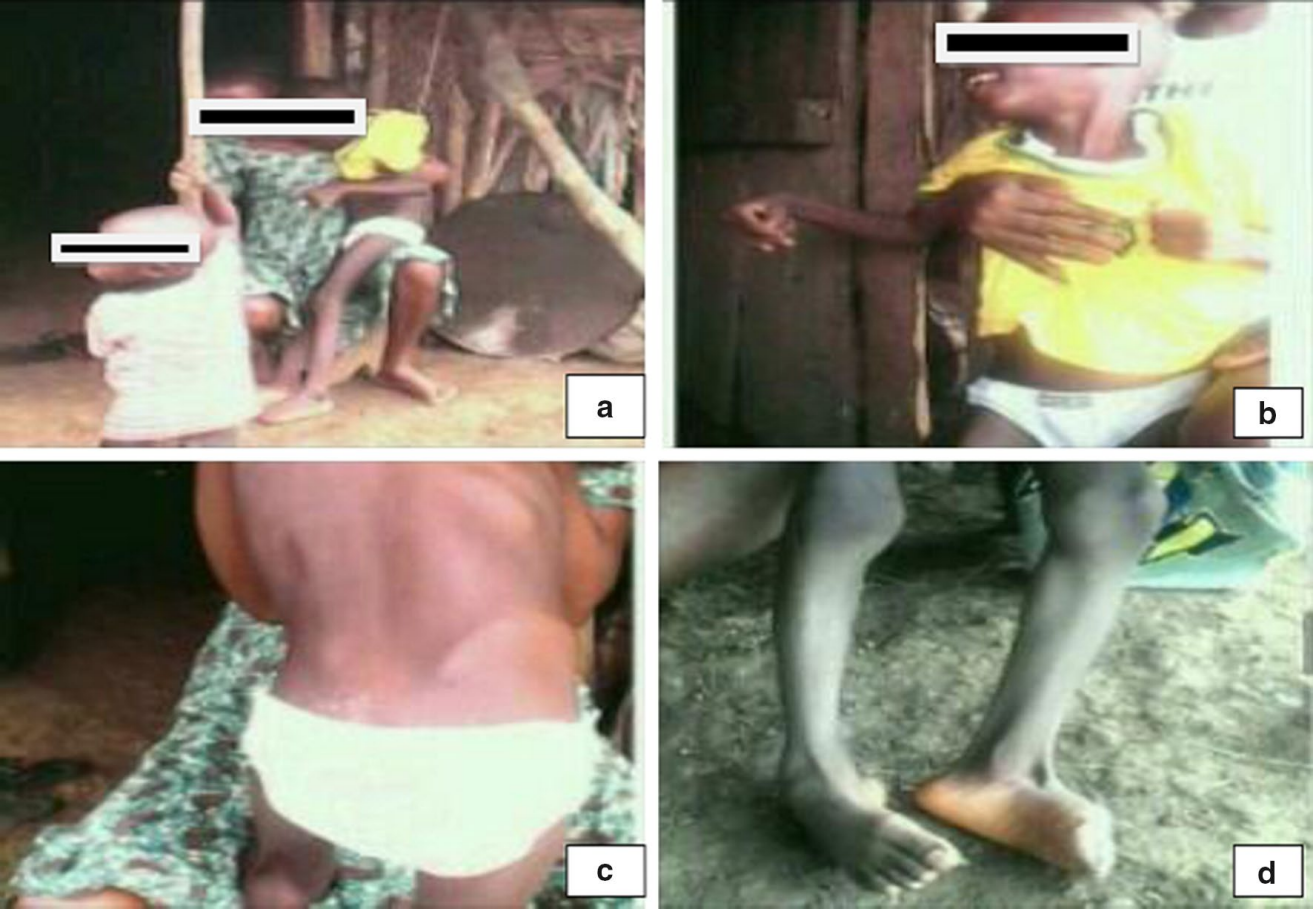
**a** Poor socio–economic condition of the community. **b** Muscle wasting and contracture of the elbow joint. **c** Wasting of the back and gluteal muscles, swaying of the hip. **d** Wasting of calf muscles and deformity of the ankle

An 8 year old male Cameroonian presented at the Grace Community Health and Development Association outreach unit with progressive muscle contractures and weakness of the lower limbs, upper limbs and trunk muscles for 4 months. The onset was marked by weakness and contractures of the lower limb, progressing within a month to involve the upper limbs and trunk muscles. This was characterized by flexural deformity of the elbow and ankle (Fig. 1b and d). These complaints were associated progressively with inability to walk or stand without support, poor head and waist control, and dysphagia. There were no seizures, cognitive impairment, visual or auditory impairment, and no bladder or bowel dysfunction. He was consulted at a district hospital where a provisional diagnosis of poliomyelitis was made. However, stool samples sent to a reference laboratory were negative for polio.

His birth history was uneventful. He stood without support at 7 months, and walked at 12 months. Assessment of other developmental mile stones was unremarkable. He had normal motor and mental functions before the onset of illness. There was no previous history of measles, HIV, trauma or hospitalization. He is fully vaccinated for age according to the national guidelines on the expanded program on immunization. There was no family history of paralytic disease or mental impairment. Both parents are alive and well.

On physical examination, the patient was conscious, afebrile, with no facial dimorphic features. Pupillary size and reflexes were normal. There was no ptosis or facial weakness. There was wasting of the upper limb, scapular, and spinal muscles and contractures of the elbows and ankles (Fig. 1c). There was no calf hypertrophy/pseudohypertrophy. Muscle power was 4/5 on both upper and lower limbs. There was weakness of the cervical, lumbar and pelvic muscles, evident by poor head control and swaying movements of the waist respectively. Hypertonia was evident on the muscles of the arm and calf, with the upper limbs placed in semi flexed position. Sensitivity to touch, pain, temperature, vibration and proprioception were preserved. Tendon and cutaneous reflexes were normal. He was unable to stand or walk without support. Gowers sign was absent. Cardiorespiratory exam was remarkable for the presence of an irregularly irregular tachycardia (heart rate was 122 beats/min). There was no palpable abdominal mass or hepatosplenomegaly. The rest of the physical examinations were unremarkable. Our differential diagnosis included Duchenne muscular dystrophy, EDMD, Fascio-scapula-humeral muscular dystrophy (FSHD), congenital myopathies, Limb-girdle muscular dystrophy (LGMD), poliomyelitis, and trichinellosis.

**Fig. 2:**
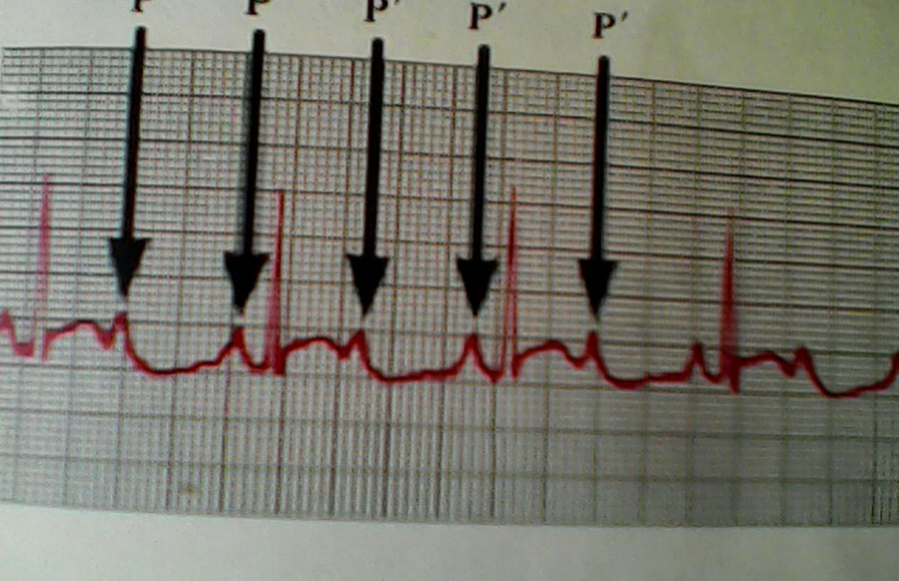
Electrocardiography showing atria tachycardia in an 8 year old male with Emery-Dreifuss muscular dystrophy in Cameroon

**Fig. 3:**
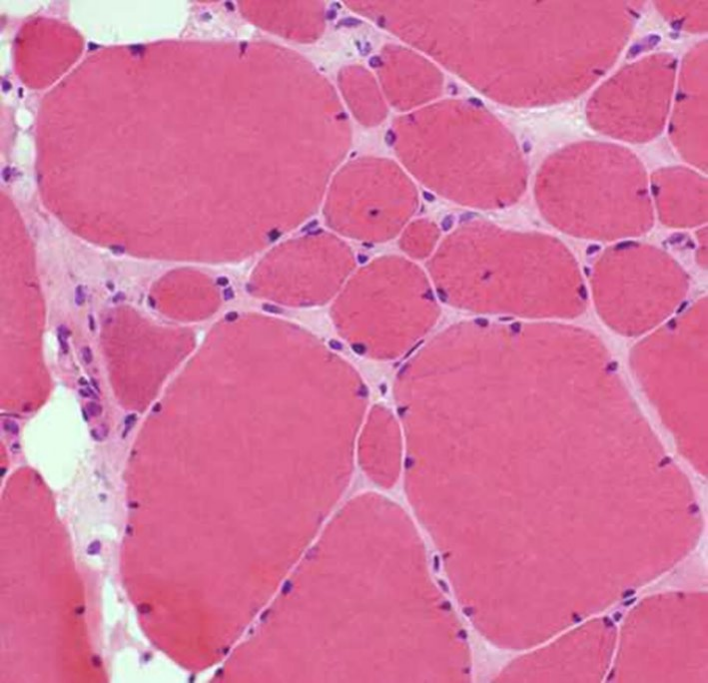
Deltoid muscle biopsy with hemoxylin–eosin staining showing variability in muscle fibres size in an 8 year old male with Emery-Dreifuss muscular dystrophy in Cameroon

Laboratory investigations showed serum creatine kinase (CK) level of 350 IU/l, hemoglobin count of 13 g/dl, white blood cell count (8700 cells/µl), and a normal metabolic profile (Table 1). Hemoxylin–eosin stain muscle biopsy analysis revealed non-specific variation of muscle fibres diameter (Fig. 2). Atrial tachycardia was evident on electrocardiography (ECG) (Fig. 3). Immuno-histological and genetic studies, and electromyography were not done due to lack of finances and diagnostic tools.

**Table 1:** Laboratory results of an 8 year old with Emery-Dreifuss muscular dystrophy in Cameroon

Investigation	Results	Reference values
Haemoglobin concentration	13 g/dl	
WBC count	8700 cells/µl	4000–10000 cells/µl
Neutrophils	4800 cells/µl	1500–7500 cells/µl
Eosinophils	2 cells/µl	1–6 cells/µl
Lymphocytes	2500 cells/µl	1200–4000 cells/µl
Platelets	250,000 cells/µl	150000–45000 cells/µl
Fasting blood sugar	98 mg/dl	65–100 mg/dl
Total cholesterol	145 mg/dl	140–199 mg/dl
Triglycerides	98 mg/dl	≤150 mg/dl
Creatine kinase (CK)	350 IU/l	170 IU/l
Stool exam	No ova, no larva	
HIV serology	Negative

A diagnosis of EDMD was made based on the early development and progression of muscle contractures, weakness and wasting with scapulohumeroperoneal pattern and cardiac arrhythmia, with atrophic changes on muscle biopsy. Expert cardiology and orthopaedic referral was done. Physiotherapy and a wheel chair were requested. Follow-up one month after referral revealed no changes in clinical outcome. Referral for expert management, physiotherapy, and purchase of wheel chair were not done because of financial constraints.

## Conclusions

Dreifuss and Hogan are credited with the initial groundwork on EDMD, when in 1961 they described an unusual X-linked form of MD in a large family with muscle wasting disease initially thought to be milder forms of Duchenne and Becker muscular dystrophies [10]. It was only in 1966 that Alan Emery and Fred Dreifuss identified EDMD as a separate disease entity [11].

Emery-Dreifuss muscular dystrophy is a rare type of muscular dystrophy with a worldwide incidence of 1 in 100,000, often presenting in late childhood and adulthood [3, 4]. It has three main genetic patterns; X-linked recessive, autosomal dominant, and autosomal recessive [5, 6]. The X-linked EDMD is the most common pattern, occurring due to mutation in the emerin gene chromosomes (Xq28) [5]. Emerin protein is located in the inner nuclear membrane of body cells, dominant in skeletal and cardiac muscles [12]. Mutation in the emerin gene causes premature termination of translation in emerin mRNA affecting protein synthesis, and causing aberration of nuclear functioning [12]. The autosomal dominant and recessive patterns of EDMD are due to mutations of the Lamin A/C genes [6]. Lamin A/C proteins forms part of the inner nuclear membrane, important in the mechanical stability of the nuclear envelop [13] and cell signalling [14].

Skeletal muscle involvement in EDMD present clinically with early contractures (involving the ankles, elbows, para-spinal muscles), weakness and wasting of scapulohumeropareoneal pattern [7, 8]. Muscles weakness may occur at the second decade and may be preceded by contractures. The main muscles affected are biceps, paravertebral, glutei, semitendinosus, adductor major, and gastrocnemius [15]. Walking difficulties and gait disturbances like in our case are common in autosomal dominant EDMD [16]. Cardiac conduction abnormalities and cardiomyopathy are the main cardiac complications of EDMD [17]. Cardiac conduction abnormalities common in EDMD include; supraventricular, and ventricular tachyarrhythmia, and atrio-ventricular block [17]. The presence of cardiac arrhythmias like in our case necessitates expert cardiologist consultation. Cardiomyopathies (more common in the autosomal dominant EDMD) [16] is a primary cause of heart failure and usually precedes cardiac arrhythmias.

Muscular dystrophies similar to EDMD include; Duchenne muscular dystrophy (DMD), Becker dystrophy (BMD), congenital muscular dystrophies, FSHD, LGMD [8]. The presence of weakness, hypotonia, and difficulty in running, jumping, or calf hypertrophy [18] should prompt investigation of DMD. However, these findings were absent in our patient. Also the scarcity of contractures in DMD [18] makes it less likely. Other possible diagnosis like congenital myopathies usually present with hypotonia and weakness at the neonatal period, which is not the case in our patient. Physical abnormalities like facial abnormalities, high palate, scoliosis, and delayed developmental mile stones are common in congenital myopathies [18], but absent in our patient. Fascio-scapula-humeral muscular dystrophy (FSHD) a dominantly inherited muscular dystrophy was a potential differential in our case. However, its association with facial weakness (ability to close the eyes tightly, or smile), slow progression of weakness, mental retardation, and absence of contractures made it less likely [18]. Limb-girdle muscular dystrophy (LGMD), another genetic form of muscular dystrophy is similar to EDMD. It predominantly causes weakness of the lower extremities. Although girdle, shoulder and neck muscle weakness can occur [18], contractures are less likely. Poliomyelitis usually presents with flaccid paralysis, making it less likely in our case.

Creatine kinase levels are normal or mildly elevated (<5 times the upper limit) in EDMD, as evident in our case. CK levels greater than 10 times the upper limit are associated with DMD, and BMD [19]. EMG is not required for diagnosis [20, 21]. Histological findings are nonspecific, displaying variability in muscle fibre diameters [20] as in our case. Molecular genetic studies are used to detect mutations of the genes. ECG and echocardiography play vital roles to diagnose cardiac complications. A team approach involving a neurologist, pulmonologist, cardiologist, orthopedic surgeon, physical therapist, and counsellors ensures quality care. Emphasis is laid on surgical and nonsurgical management of contractures, and pacemaker implantations to prevent sudden death from cardiac complications [21].

Our case emphasizes the importance of community outreach programs in the recognition of rare diseases like EDMD, as this was the historical background in the discovery of this disease [10]. Although EDMD has no definitive treatment, early diagnosis and interventions play an important role in improving the wellbeing of the patients [22]. Early recognition of EDMD helps in minimizing contractures through early physical therapy and stretching exercises [22]. However, this was not the case in our patient as the contractures had markedly progressed resulting in the patient being handicapped and consequently bed bound. Also the presence of cardiac signs on ECG puts the patient at risk of sudden death from cardiac complications like arrhythmias and other cardiac myopathies. Early recognition of these cardiac complications is required for timely cardiac interventions like pacemaker implantation to prevent sudden cardiac death [22]. Genetic and clinical screening of family members is also warranted as it plays an important role in early recognition of family members at risk [22]. However, genetic studies were not done in our case because of limited resources. Furthermore, scientific research in recognizing the genes, mutations, and risk factors associated with EDMD should be encouraged in the development of novel gene therapies in the management of EDMD. The development and support of organizations involved in the recognition, awareness and management of muscular dystrophies in resource limited settings will play a good role in finding and assisting poor children with EDMD.

Emery-Dreifuss muscular dystrophy is a rare disabling genetic muscular disease, and a cause of paralysis, and sudden death from cardiac complications. A high index of suspicion is required, to pose a diagnosis in patients with paralysis in resource limited settings. Prompt diagnosis and management is essential to prevent the progression of cardiac and orthopedic complications and improve the prognosis of this disease.

## Abbreviations

BMD: Becker muscular dystrophy
DMD: Duchenne muscular dystrophy
EDMD: Emery-Dreifuss muscular dystrophy
FSHD: Fascio-scapula-humeral muscular dystrophy
LGMD: Limb-girdle muscular dystrophy
MD: muscular dystrophy
